# Dual nature of ferritin for hematologic, liver functional, and metabolic parameters in older diabetic patients

**DOI:** 10.1038/s41598-023-47678-5

**Published:** 2023-11-18

**Authors:** Jui-Hua Huang, Ren-Hau Li, Leih-Ching Tsai

**Affiliations:** 1 Department of Golden-Ager Industry Management, Chaoyang University of Technology, Taichung, 413 Taiwan; 2 Department of Psychology, Chung Shan Medical University, Taichung, 402 Taiwan; 3 Division of Endocrine and Metabolism, Department of Internal Medicine, Erlin-Branch, Changhua Christian Hospital, Changhua, Taiwan

**Keywords:** Diseases, Health care, Risk factors

## Abstract

This study explored the association between ferritin with hematologic, liver functional, and metabolic parameters in older diabetic patients. A total of 210 diabetic patients aged 65 or older were classified into four groups according to the reference range of serum ferritin. Demographic variables and health-related lifestyle factors were obtained through the utilization of a standardized questionnaire. Anthropometric measures, blood pressure, hematology test, and biochemical assessment were also performed**.** Among all patients, 29.5% had anemia. The percentage of anemia in groups low ferritin (< 40 μg/L), lower side within the reference range (40–120 μg/L), higher side within the reference range (121–200 μg/L), and high ferritin levels (> 200 μg/L) were 50.0, 27.7, 20.5, and 24.2% (*P* = 0.025), respectively. Low ferritin levels had a higher risk of anemia and a high red blood cell distribution width (RDW). High ferritin levels were associated with a higher risk of high glutamate pyruvate transaminase, obesity, high fasting blood glucose (FBG), and high postprandial blood glucose. The higher side within the reference range of ferritin also showed a higher risk of high FBG and high glycated hemoglobin. Nevertheless, there was no significant association between ferritin and inflammation marker, serum lipids or blood pressure. Overall, ferritin demonstrates a dual nature in older diabetic patients: low ferritin levels are linked to anemia or elevated RDW, while high levels are linked to obesity, increased liver enzymes, and worse glycemia control.

## Introduction

Diabetes is a significant public health issue in the aging population. Globally, 19.3% of 65–99-year-olds have diabetes in 2019^1^. Approximately 29.2% of the US population aged 65 or older had diabetes diagnosed in 2019^2^. In Taiwan, the diabetes prevalence of adults above 65 in 2017–2020 was 25.4%^3^. Diabetes-related complications are more common in older adults than in young people^4^. Variability in glucose, blood pressure, plasma lipids, body weight, and other organ functions are risk factors for diabetes-related complications^5^. It is worth noting that iron stores, expressed as serum ferritin, are associated with these risk factors for diabetic glycemia control and complications^6–8^.

A protein called ferritin plays an important role in iron homeostasis, and its levels can be used as an indirect indicator of iron storage^9^. Nevertheless, both too low and too high levels of ferritin may have implications for patients with diabetes^7^. Low ferritin levels can indicate iron deficiency anemia, which is prevalent in older adults^10^. Anemia may result in fatigue, weakness, and shortness of breath^11^. These symptoms can affect an older person's ability to manage their diabetes effectively. Additionally, temporal iron deficiency is related to sensitized insulin action, but chronic iron deficiency with anemia can increase the risk of cardiovascular diseases in diabetic patients^12^. Consequently, the potential influence of low ferritin levels on hematological alterations and their contribution to complications associated with diabetes is noteworthy. However, reports are scarce on the correlation between low ferritin levels and hematological parameters in older patients with diabetes.

In several systemic diseases, iron metabolism is closely related to clinical manifestations^8^. A high serum ferritin level can be a sign of inflammation or iron overload, both of which can negatively affect patients with diabetes^8,12^. Chronic inflammation can contribute to insulin resistance, which may influence glucose metabolism^13^. Additionally, iron overload increases the risk of cardiometabolic disorders and liver dysfunction^14–16^. Excessive iron can impair insulin secretion and increase insulin resistance, which is related to obesity and type 2 diabetes^7,13,17^. As a result, iron overload may be a contributing factor to the onset of insulin resistance and associated organ damage. However, the association between elevated serum ferritin levels and glucose metabolism, cardiometabolic risk factors, and liver function in older diabetic patients is still unclear.

There is growing evidence that iron metabolism is affected by the aging process. Iron deficiency anemia is common in older populations, and Hb levels also decline with advancing age^18^. Nevertheless, serum ferritin alterations are not a necessary result of aging^19^. Ferritin is an iron-storing protein. Its levels are influenced not only by how much iron is in the body but also by inflammation^9,14,20^. Aging is an inflammatory state, and type 2 diabetes is a disease related to aging, obesity, and inflammation^17,21,22^. However, the correlation between bodily iron reserves and hematologic parameters, organ function, and diabetes control in older diabetic patients remains indeterminate. The objective of this investigation was to examine the correlation between serum ferritin levels and the presence of anemia, liver dysfunction, and metabolic abnormalities in older diabetic patients. In addition to demographic data and lifestyle habits, anthropometric measurements, blood pressure, hematology tests, and biochemical assessments were conducted.

## Methods

### Ethical approval

The Institutional Review Board of Changhua Christian Hospital in Taiwan granted approval for this study (CCHIRB No: 090419) and conducted according to the Declaration of Helsinki. In addition, informed consent was obtained from all participants involved in the study.

### Study sample

Based on cross-sectional studies/surveys, this study's sample size was estimated^23^.

Taiwan’s elderly populations may have anemia prevalence between 10.5 and 18.8%, according to previous studies^24,25^. We calculated a sample size between 144 and 235 based on a precision/absolute error of 5% and an error type 1 of 5%.

In addition, this study performed the chi-square Goodness of Fit test using the data of the distribution ratio of the male (n = 244, 51.4%) and female (n = 231, 48.6%) population of the 475 people with diabetes aged 65 years or over from 2017 to 2020 of Nutrition and Health Survey in Taiwan (NAHSIT)^3^ and the number of male (n = 94, 46.6%) and female (n = 112, 53.3%) of the 210 samples in this study. According to the chi-square Goodness of Fit test, the *P* value was 0.170, which did not reach statistical significance. It is obvious that the sample of this study should be quite representative. The results of this study can be extrapolated to the whole Taiwan.

After informed consent was obtained, this study recruited 236 elderly patients with type 2 diabetes aged 65 years or older from central Taiwan. Type 2 diabetes for more than six months, no change in medication for the past three months, and a stable lifestyle were included in the study. There was exclusion for major illness, infections, acute hemorrhage, thalassemia, and signs of severe cognitive decline. The study excluded 26 elderly patients with type 2 diabetes who did not provide adequate information regarding their personal data, lifestyle habits, and health status, and 210 older diabetes patients participated in the study.

### Assessment of covariables for demographic variables and lifestyle-related habits

Information on demographic variables and lifestyle habits, including dietary intake, smoking status, alcohol intake, and physical activity level, was collected using a standardized questionnaire. Dietary intake was assessed using a 24-h recall and a typical nutritional pattern for a week. Based on the Taiwan Nutrition Database, nutritional analysis software (Professional Edition, 2001/2003, EKitchen Inc., Taichung, Taiwan) was used to estimate energy, protein, fat, carbohydrate, and iron intake. Insufficient intakes of Fe are defined as Fe intakes below dietary reference intakes *(*DRIs) for Fe for Taiwanese aged 65 years and older (10 mg/day)^26^.

This study estimated total physical activity by using a Finnish method^27^. In order to estimate total physical activity, participants reported three types of physical activity: occupational, commuting, and leisure-time physical activity. The total physical activity was further evaluated and classified into three categories: low, moderate, and high.

Additionally, we collected information about smoking habits and alcohol consumption. The study classified smoking habits into three categories: never smoked, formerly smoked, currently smoking, and alcohol consumption, which was classified as never consumed, previously consumed, and currently consumed.

### Measurement of ferritin and hematologic, nutritional, inflammatory, liver functional, and metabolic parameters

The participants were asked to fast for 8 to 10 h before the blood was measured. The hospital medical laboratory (certified ISO 15189) measured ferritin and inflammatory, nutritional, hematologic, liver functional, and metabolic parameters to assess iron store status and risk of inflammation, anemia, liver dysfunction, and metabolic abnormality.

The ferritin, hemoglobin (Hb), red blood cell distribution width (RDW), and mean corpuscular volume (MCV) were measured using a hematology analyzer (Beckman Coulter**,** Fullerton, California, USA). The detection of platelets using a fully automated hematology analyzer (XT1800i, Sysmex, Kobe-shi, Hyogo, Japan). Serum albumin, transferrin, high-sensitivity C-reactive protein (hsCRP), glutamate pyruvate transaminase (GPT), glutamate oxaloacetate transaminase (GOT), glucose, and lipids were measured using a Dimension RxL autoanalyzer (Siemens, Newark, USA).

### Serum ferritin determination

Ferritin was measured by a two-site immunoenzymatic (“sandwich”) assay. Ferritin blood tests were used to determine iron levels in the body. Based on a reference range of 40–200 μg/L^28–30^, serum ferritin levels were divided into four subgroups: 1) low ferritin level was below 40 μg/L; 2) lower side within the reference range was 40–120 μg/L; 3) higher side within the reference range was 121–200; and 4) high ferritin level was over 200 μg/L.

### Inflammatory marker determination

For the detection of inflammation, a high-sensitivity C-reactive protein (hs-CRP) test is used. Using a particle-enhanced turbidimetric immunoassay, the hsCRP was determined. In terms of forecasting the risk of cardiovascular disease, the laboratory’s clinical significance states that hsCRP levels less than 1 mg/L are low risk, 1 to 3 mg/L are moderate risk, and more than 3 mg/L are high risk. A result of more than 10 mg/L for hsCRP indicates the presence of an infection or another acute cause of inflammation^31^.

### Hematologic and nutritional parameter determination

An assessment of nutrition status is based on the measurement of serum albumin and transferrin. Serum albumin was estimated using the bromcresol purple method. The turbidimetric method was used to estimate serum transferrin. In addition, Hb, RDW, and MCV were measured by the automated cell count. Diagnosis of anemia by Hb test. The World Health Organization (WHO) defines anemia as Hb levels below 12 g/dL in females and 13 g/dL in males^32^. The RDW serves as a customary component of the comprehensive blood count and serves as an indicator of the heterogeneity in the size of red blood cells^33^. As per the medical laboratory of the hospital, the RDW reference range was determined to be 11.7 ~ 14.9%. The platelets were determined by the direct current detection method. An evaluation of the platelet count in the blood to detect thrombocytes^34^.

### Liver functional parameter determination

The GPT and GOT were used to determine liver function. The GPT and GOP were measured by UV/NADH (rate method). According to the hospital medical laboratory, the reference range of GPT was 11 ~ 40 U/L. The reference range of the GOP was 15 ~ 41 U/L.

### Metabolic parameter determination

The calculation of body mass index (BMI) involved the division of weight in kilograms by the square of height in meters [(kg)/(m^2^)]. The Health Promotion Administration, Ministry of Health and Welfare in Taiwan, defines obesity as having a body mass index (BMI) greater than 27 kg/m^2^^35^. Assessment of body fat percentage by performing bioelectrical impedance analysis using TBF-410 (TANITA, Tokyo, Japan). Females with a body fat percentage exceeding 30% and men with a body fat percentage greater than 25% were classified as obese. Furthermore, the recommended target for managing blood pressure is a reading below 140/90 mmHg^36^.

Measuring glycated hemoglobin (HbA1c), fasting blood glucose (FBG), and postprandial blood glucose (PBG) to assess glycemic control. The HbA1C was measured by ion-exchange chromatography (BioRad Variant, Hercules, California, USA). The FBG and PBG were measured by hexokinase-UV/NAD (end reaction) methods. The glycemic goals recommended for older adults include HbA1c≦7.5%, FBG≦130 mg/dL, and PBG≦180 mg/dL. Conversely, glycemic control is deemed inadequate when HbA1c > 7.5%, FBG > 130 mg/dL, and PBG > 180 mg/dL are observed^36^. In addition, assessment of lipid levels in the blood, including high-density lipoprotein cholesterol (HDL-C) and triglyceride. The HDL and triglyceride were assessed using the timed-endpoint method and standard enzymatic methods, respectively. Elevated triglyceride levels during fasting that are 150 mg/dL or higher. Low HDL-C is defined as having values of less than 40 mg/dL in men and less than 50 mg/dL in women^37^.

### Statistical analysis

The Chi-square test is used to investigate the correlation between two categorical variables. Data are numbers (n), percentages (%). The means of two groups were compared using a t-test. The data are means ± standard deviations. Correlations between serum ferritin with hematologic, liver functional and metabolic parameters were examined by adjusted simple correlation. Data are correlation coefficient (r). A one-way analysis of variance (ANOVA) was used to compare the means of four groups, followed by a Bonferroni's multiple comparison test. Conducting ANOVA trend analyses using line by polynomial contrasts after a comparison was made between the means of four groups. Data are means ± SD. A logistic regression model was used to investigate the correlation between serum ferritin levels and the likelihood of developing hematological abnormalities, liver dysfunction, and metabolic risk factors. The confounding factors that were adjusted for included sex, age, medication for diabetes, dietary intake, levels of physical activity, smoking, alcohol consumption, and hsCRP. Data are odds ratios (95% CIs). The results were found to be statistically significant with a *p*-value of less than 0.05.

## Results

### Characteristics of participants based on serum ferritin levels

Characteristics of the 210 older patients with type 2 diabetes by serum ferritin levels were showed in Table 1. No significant differences were observed in age, gender, diabetes medication, dietary intake, physical activity levels, smoking, alcohol consumption, and hsCRP among the four subgroups.

**Table 1:** Characteristics of participants based on serum ferritin levels.

Variables	Serum ferritin levels (μg/L)				*p*
	Low	Lower side within reference range	Higher side within reference range	High	
	< 40	40–120	121–200	> 200	
	n = 36	n = 101	n = 39	n = 33	
Age (y)	72.4 ± 5.0	72.1 ± 5.4	72.4 ± 5.6	72.5 ± 5.2	0.970
Gender					
Male	19 (52.8)	41 (40.6)	19 (48.7)	18 (54.5)	0.404
Female	17 (47.2)	60 (59.4)	20 (51.3)	15 (45.5)	
Diabetes medication					
Oral hypoglycemic drug	26 (72.2)	72 (71.3)	23 (59.0)	25 (75.8)	0.402
Insulin and Oral hypoglycemic drug	10 (27.8)	29 (28.7)	16 (41.0)	8 (24.2)	
Dietary intake					
Total energy intake (kcal/day)	1660 ± 383	1547 ± 366	1657 ± 537	1536 ± 419	0.303
Protein intake (% of energy)	12.3 ± 2.3	12.8 ± 2.7	11.8 ± 2.2	12.0 ± 3.0	0.200
Carbohydrate intake (% of energy)	59.7 ± 7.9	60.6 ± 8.4	61.7 ± 8.2	61.8 ± 9.9	0.677
Fat intake (% of energy)	27.1 ± 6.8	26.7 ± 7.3	26.2 ± 7.5	26.5 ± 8.1	0.971
Fe intake (mg/day)	10.6 ± 5.3	10.1 ± 7.3	8.5 ± 5.3	9.2 ± 6.2	0.464
< 10 mg/day	20 (55.6)	65 (64.4)	28 (71.8)	21 (63.6)	0.541
Physical activity levels					
Low	6 (16.7)	23 (22.8)	9 (23.1)	10 (30.3)	0.394
Moderate	19 (52.8)	40 (39.6)	11 (28.2)	12 (36.4)	
High	11 (30.6)	38 (37.6)	19 (48.7)	11 (33.3)	
Smoking					
Never smoked	25 (69.4)	72 (71.3)	25 (64.1)	26 (78.8)	0.868
Formerly smoked	6 (16.7)	19 (18.8)	9 (23.1)	5 (15.2)	
Currently smoking	5 (13.9)	10 (9.9)	5 (12.8)	2 (6.1)	
Alcohol consumption					
Never consumed	29 (80.6)	88 (87.1)	31 (79.5)	28 (84.8)	0.639
Formerly consumed	3 (8.3)	7 (6.9)	5 (12.8)	1 (3.0)	
Currently consuming	4 (11.1)	6 (5.9)	3 (7.7)	4 (12.1)	
Inflammatory marker: hsCRP (mg/L)	3.0 ± 3.6	2.6 ± 3.9	2.7 ± 3.0	2.1 ± 3.2	0.670
Anemia					
With	18 (50.0)	28 (27.7)	8 (20.5)	8 (24.2)	0.025
Without	18 (50.0)	73 (72.3)	31 (79.5)	25 (75.8)	

The Chi*-*square test is used to examine association between two categorical variables*.* Data are number (n), percent (%). The means of four groups were compared using one-way ANOVA. Data are means ± SD. Significant difference (*p* < 0.05).

hsCRP, high-sensitivity C-reactive protein; Hb, hemoglobin.

Anemia is defined as Hb < 12 g/dL for females and < 13 g/dL for males.

In addition, among all patients, 64.3% had Fe intake insufficient, 29.5% had anemia, 17.1% had low serum ferritin, 9.5% had anemia and low serum ferritin. The study found that among elderly diabetic patients, the percentage of anemia varied across different serum ferritin levels, with rates of 50.0, 27.7, 20.5, and 24.2% (*p* = 0.025) observed in the low, lower side within reference range, higher side within reference range, and high serum ferritin groups, respectively.

### Adjusted simple correlations between ferritin and hematologic, liver functional, and metabolic parameters

Adjusted simple correlation was performed to examine the simple correlations between serum ferritin and hematologic, liver functional, and metabolic parameters. Adjusted factors were sex, age, diabetes medication, dietary intake, physical activity levels, smoking, and alcohol consumption. A significant positive relationship was found between the ferritin and serum albumin (r = 0.153; *p* = 0.031) and Hb (r = 0.169; *p* = 0.017). Ferritin is also positively correlated with GPT (r = 0.202; *p* = 0.004), GOT (r = 0.150; *p* = 0.034), BMI (r = 0.147; *p* = 0.038), body fat percentage (r = 0.156; *p* = 0.027), HbA1c (r = 0.174; *p* = 0.014), FBG (r = 0.251; *p* =  < 0.001), and PBG (r = 0.212; *p* = 0.003).

In addition, ferritin was negatively correlated with RDW (r = − 0.234; *p* = 0.001) and platelets (r = − 0.253; *p* < 0.001). Ferritin was not significantly correlated with transferrin, MCV, triglyceride, HDL-C, systolic BP, and diastolic BP.

### Mean of nutritional and hematologic, liver functional, and metabolic parameters by serum ferritin levels

As shown in Table 2, A one-way ANOVA was performed to investigate the disparities among the means of four distinct subgroups. The results indicated statistically significant variations in the levels of Hb (*p* = 0.010), RDW (*p* = 0.006), MCV (*p* = 0.009), platelet (*p* = 0.001), GOT (*p* = 0.024), GPT (*p* = 0.005), BMI (*p* = 0.002), body fat (*p* = 0.047), HbA1c (*p* = 0.015), FBG (*p* = 0.040) and PBG (*p* = 0.025) across the four subgroups. Results of Bonferroni post hoc test showed that a low level of serum ferritin showed a significantly lower Hb than that of higher side within reference range and high serum ferritin subgroups. Moreover, a low level of ferritin showed a significantly higher RDW than a high level of serum ferritin. A high level of serum ferritin subgroup showed a significantly higher MCV than a higher side within the reference range subgroup. The low, lower side within the reference range and the higher side within reference range subgroup showed significantly higher platelets compared with a high level of ferritin, especially a low serum ferritin level. Furthermore, the results indicate that a high level of ferritin is associated with a significantly higher GOP compared to the lower side within the reference range of serum ferritin. Similarly, a high level of ferritin is associated with a significantly higher GPT compared to both low and lower sides within the reference range of serum ferritin. Additionally, a high level of ferritin was associated with a significantly higher BMI and PBG when compared to a low level of ferritin. Moreover, a higher side within the reference range of serum ferritin is associated with a significantly higher HbA1c compared to a low level of serum ferritin. However, no significant association was found between ferritin and hsCRP, triglycerides, HDL-cholesterol or blood pressure.

**Table 2:** Mean of nutritional and hematologic parameters, liver functional parameters and metabolic parameters by serum ferritin levels.

Variables	Serum ferritin levels (μg/L)				ANOVA F, *P*	*P* for trend
	Low	Lower side within the reference range	Higher side within the reference range	High		
	< 40	40–120	121–200	> 200		
	n = 36	n = 101	n = 39	n = 33		
Nutritional and hematologic parameters						
Albumin (g/dL)	3.97 ± 0.29	3.98 ± 0.29	3.99 ± 0.33	4.07 ± 0.27	0.867, 0.459	0.169
Transferrin (mg/dL)	263.9 ± 56.8	245.7 ± 36.4	246.3 ± 37.6	244.4 ± 40.3	1.951, 0.123	0.063
Hb (g/dL)^a^^,^^b^	12.4 ± 1.7	13.1 ± 1.5	13.3 ± 1.4	13.5 ± 1.6	3.859, 0.010	0.001
RDW (%)^b^	13.9 ± 1.1	13.5 ± 1.1	13.6 ± 1.0	13.0 ± 0.9	4.228, 0.006	0.001
MCV (fL)^d^	87.5 ± 7.6	89.1 ± 6.3	86.1 ± 8.1	91.5 ± 6.8	3.922, 0.009	0.089
Platelet (10^3^/uL)^b,c,d^	227.6 ± 59.3	217.5 ± 52.4	213.0 ± 51.5	175.6 ± 62.2	6.097, 0.001	< 0.001
Liver functional parameters						
GOP (U/L)^c^	24.7 ± 12.8	25.6 ± 12.4	25.1 ± 12.6	34.3 ± 26.0	3.209, 0.024	0.015
GPT (U/L)^b^^,^^c^	39.2 ± 16.6	45.0 ± 17.9	47.1 ± 20.9	56.2 ± 27.8	4.430, 0.005	< 0.001
Metabolic parameters						
BMI (kg/m^2^)^b^	23.9 ± 3.9	25.7 ± 3.6	24.5 ± 3.1	26.8 ± 3.9	4.944, 0.002	0.005
Body fat (%)	25.2 ± 9.1	28.5 ± 8.9	28.8 ± 9.9	30.6 ± 9.1	2.701, 0.047	0.028
HbA1c (%)^a^	6.9 ± 0.9	7.2 ± 1.1	7.9 ± 1.6	7.5 ± 1.7	3.559, 0.015	0.017
FBG (mg/dL)	122.4 ± 36.0	127.4 ± 40.1	142.6 ± 34.3	142.7 ± 49.5	2.825, 0.040	0.012
PBG (mg/dL)^b^	160.6 ± 47.6	184.3 ± 58.7	190.2 ± 69.6	206.2 ± 77.4	3.183, 0.025	0.003
Triglycerides (mg/dL)	95.1 ± 58.7	106.9 ± 63.9	116.9 ± 56.0	104.6 ± 44.5	0.886, 0.460	0.381
HDL-cholesterol (mg/dL)	48.6 ± 15.4	47.2 ± 11.0	46.2 ± 13.7	45.2 ± 10.4	0.505, 0.679	0.221
Systolic BP (mm Hg)	135.5 ± 13.2	135.8 ± 15.8	136.9 ± 15.0	132.4 ± 13.0	0.582, 0.627	0.465
Diastolic BP (mm Hg)	77.6 ± 13.1	76.8 ± 14.3	77.5 ± 12.3	77.7 ± 11.9	0.063, 0.979	0.929

The means of four groups were compared using one-way ANOVA followed by Bonferroni’s multiple comparisons test. Conducting ANOVA trend analyses using line by polynomial contrasts after a difference between the means of four groups were compared. Data are means ± SD. Statistically significant at *p* < 0.05.

^a^Indicates significant difference between ferritin levels < 40 μg/L and 121–200 μg/L.

^b^Indicates significant difference between ferritin levels < 40 μg/L and > 200 μg/L.

^c^Indicates significant difference between ferritin levels 40–120 μg/L and > 200 μg/L.

^d^Indicates significant difference between ferritin levels 121–200 μg/L and > 200 μg/L.

Hb, hemoglobin; RDW, red blood cell distribution width; GPT, Glutamate pyruvate transaminase; GOT: Glutamate oxaloacetate transaminase; BMI, body mass index; HbA1C, glycated hemoglobin; FBG: Fasting blood glucose; PBG: postprandial blood glucose; BMI: Body mass index; HDL, high-density lipoprotein; BP: blood pressure.

On the other hand, results of conducting ANOVA trend analyses showed that the transferrin levels exhibited a slight decrease in correspondence with an elevation in serum ferritin levels (*p* for trend = 0.063). For hematologic parameters, the hemoglobin levels exhibited a significant increase in correlation with elevated serum ferritin levels. (*p* for trend = 0.001). The results indicate a statistically significant decrease in RDW (*p* for trend = 0.001) and platelet count (*p* for trend < 0.001) with an increase in serum ferritin levels. For liver functional parameters, GOP (*p* for trend = 0.015) and GPT (*p* for trend < 0.001) were significantly higher with increasing serum ferritin levels. For metabolic parameters, BMI (*p* for trend = 0.005), body fat percentage (*p* for trend = 0.028), HbA1c (*p* for trend = 0.017), FBG (*p* for trend = 0.012), and PBG (*p* for trend = 0.003) were significantly higher with increase of serum ferritin levels.

### Mean serum ferritin by hematologic, liver functional, and metabolic parameters status

Table 3 shows the mean serum ferritin by hematologic parameters, liver functional parameters, and metabolic parameters status. Mean ferritin levels were significantly lower in patients with high RDW (*p* = 0.038). Additionally, ferritin levels were significantly higher in patients with high GPT (*p* = 0.007), high body fat percent (*p* = 0.022), high FBG (*p* = 0.012), and high PBG (*p* = 0.028). Moreover, the mean serum ferritin is approximately between 126.8 μg/L and 135.2 μg/L among patients with high GPT, high body fat percent, high FBG, and high PBG. However, there was no statistically significant variance in the average serum ferritin levels among patients with and without anemia, elevated GOP, obesity (BMI > 27 kg/m^2^), and elevated HbA1c.

**Table 3:** Mean **s**erum ferritin by hematologic parameters, liver functional parameters and metabolic parameters status.

Variables	n	Serum ferritin levels (μg/L)	*p*
Hematological parameters			
Anemia^3^			
With	62	104.0 ± 105.6	0.221
Without	148	121.4 ± 88.5	
RDW > 14.9%			
With	25	79.8 ± 59.9	0.038
Without	185	121.2 ± 96.7	
Liver function parameters			
GOP > 40 U/L			
With	27	138.6 ± 99.5	0.187
Without	183	113.0 ± 93.0	
GPT > 41 U/L			
With	88	137.9 ± 109.5	0.007
Without	122	100.7 ± 77.7	
Metabolic parameters			
BMI > 27 kg/m^2^ (obese)			
With	59	133.0 ± 108.8	0.106
Without	151	109.7 ± 87.0	
Body fat: Male > 25% and female > 30% (obese)			
With	109	130.6 ± 100.1	0.022
Without	101	100.9 ± 84.6	
HbA1c > 7.5%			
With	63	127.5 ± 98.0	0.260
Without	147	111.5 ± 82.1	
FBG > 130 mg/dL			
With	89	135.2 ± 106.6	0.012
Without	121	102.3 ± 81.1	
PBG > 180 mg/dL			
With	107	130.2 ± 105.0	0.028
Without	103	101.8 ± 78.9	

The means of two groups were compared using t-test. Data are means ± SD. Significant difference (*p* < 0.05).

Hb, hemoglobin; RDW, red blood cell distribution width; GPT, Glutamate pyruvate transaminase; GOT: Glutamate oxaloacetate transaminase; BMI, body mass index; HbA1C, glycated hemoglobin; FBG: Fasting blood glucose; PBG: postprandial blood glucose.

Anemia is defined as Hb < 12 g/dL for females and < 13 g/dL for males.

### Odds ratio of hematological abnormalities, liver dysfunction, and metabolic risk factors by serum ferritin levels

Table 4 shows the adjusted OR of hematological abnormalities, liver dysfunction and metabolic risk factors according to serum ferritin levels. After adjusting for sex, age, diabetes medication, dietary intake, physical activity levels, smoking, alcohol consumption, and hsCRP, a low level of ferritin demonstrated a significantly higher risk of anemia (OR 3.47) and high RDW (OR 10.41) in comparison to a high level of ferritin.

**Table 4:** Odds ratio of abnormal hematological indices, liver dysfunction, and metabolic abnormality by serum ferritin levels.

Variables^1^	Serum ferritin levels (μg/L)			
	Low	Lower side within the reference range	Higher side within the reference range	High
	< 40	40–120	121–200	> 200
	n = 36	n = 101	n = 39	n = 33
Abnormal hematological indices				
Anemia^2^	3.47 (1.13–10.64)	1.30 (0.50–3.40)	0.75 (0.23–2.46)	1.00
*p*	0.029	0.589	0.635	
RDW > 14.9%	10.41 (1.06–102.59)	5.25 (0.59–47.08)	6.83 (0.65–71.46)	1.00
*p*	0.045	0.138	0.109	
Liver dysfunction				
GOP > 40 U/L	1.00	1.27 (0.31–5.26)	1.74 (0.35–8.77)	3.29 (0.70–15.53)
*p*		0.742	0.500	0.132
GPT > 41 U/L	1.00	3.61 (1.36–9.60)	3.53 (1.15–10.80)	7.88 (2.44–25.52)
*p*		0.010	0.027	0.001
Metabolic abnormality				
BMI > 27 kg/m^2^ (obese)	1.00	3.01 (0.92–9.83)	2.56 (0.67–9.77)	4.80 (1.25–18.47)
*p*		0.069	0.170	0.022
Body fat: Male > 25% and female > 30% (obese)	1.00	3.49 (1.31–9.30)	5.77 (1.82–18.32)	12.92 (3.40–49.06)
*p*		0.012	0.003	< 0.001
HbA1c > 7.5%	1.00	2.69 (0.84–8.64)	6.66 (1.85–23.89)	3.24 (0.83–12.60)
*p*		0.096	0.004	0.090
FBG > 130 mg/dL	1.00	2.29 (0.89–5.90)	4.39 (1.49–12.92)	4.28 (1.39–13.24)
*p*		0.085	0.007	0.012
PBG > 180 mg/dL	1.00	1.34 (0.56–3.17)	1.22 (0.44–3.38)	4.01 (1.29–12.45)
*p*		0.511	0.703	0.016

Logistic regression models were used to examine the relationship between serum ferritin level (as a predictor variable) with hematologic, liver functional and metabolic risk factors (as a dependent variable, respectively). Adjusted confounding factors were sex, age, diabetes medication, dietary intake, physical activity levels, smoking, alcohol consumption, and HsCRP. Data are odds ratios (95% CIs). Statistically significant at p < 0.05.

^1^Hb, hemoglobin; RDW, red blood cell distribution width; GOT, Glutamate oxaloacetate transaminase; GPT, Glutamate pyruvate transaminase; BMI, body mass index; HbA1C, glycated hemoglobin; FBG, Fasting blood glucose; PBG, postprandial blood glucose; BMI, Body mass index.

^2^Anemia is defined as Hb < 12 g/dL for females and < 13 g/dL for males.

In comparison to a low level of ferritin, the lower and higher side within the reference range, as well as high serum ferritin, were found to be significantly associated with an increased risk of high GPT. This association was particularly strong for high serum ferritin, with an odds ratio of 7.88. The high serum ferritin level showed a significantly higher risk of obesity (BMI > 27 kg/m^2^, OR 4.80). Lower side within the reference range, higher side within the reference range and high serum ferritin showed a significantly higher risk of obesity (body fat: Male > 25% and female > 30), especially high serum ferritin (OR 12.92). The higher side within reference range of serum ferritin showed a significantly higher risk of high HbA1c (OR 6.66). Additionally, individuals with higher side within the reference range and high ferritin levels were found to have a significantly increased risk of high FBG, as indicated by OR of 4.39 and 4.28, respectively. A statistically significant association was observed between high serum ferritin levels and an increased risk of high PBG (OR 4.01). However, no significant association was found between serum ferritin levels and GOP.

## Discussion

This cross-sectional study aimed to determine if serum ferritin levels are correlated with hematologic alterations, liver function, and metabolic risk factors among elderly patients diagnosed with type 2 diabetes. The findings of the present study indicate that (1) about 29.5% of elderly diabetes patients had anemia; (2) low serum ferritin (< 40 μg/L) was associated with a risk of anemia and high RDW; (3) high serum ferritin (> 200 μg/L) showed a significantly higher risk of high GPT; (4) the higher side within the reference range (121–200 μg/L) and high serum ferritin (> 200 μg/L) were related to liver dysfunction, obesity, and poor glycemic control.

### Relationship between aging, diabetes and anemia

Diabetes is a highly prevalent public health problem in the aging population. The prevalence of anemia also demonstrated a positive correlation with advancing age^38,39^. The coexistence of diabetes and anemia is common in the elderly population^40,41^. Furthermore, research has demonstrated that individuals diagnosed with type 2 diabetes exhibit a greater prevalence of anemia in comparison to those without the condition^42^. Among people with type 2 diabetes between the ages of 18 and 65, anemia prevalence was 17.9% in a cross-sectional study^43^. A study population of 245 diabetic patients aged ≥ 60, the prevalence of anemia was found in 21.6%^44^. According to a cross-sectional study, the older population exhibited a prevalence rate of 35.3% for anemia, while the prevalence rate for anemia among older individuals with diabetes was 38.6%^42^. Several studies have indicated that the prevalence of anemia among elderly individuals in Taiwan ranges from 10.5% to 18.8%^24,25^. The present study revealed a percentage of approximately 29.5% for anemia among elderly patients diagnosed with type 2 diabetes.

The prevalence of anemia in older people with diabetes ranges from 21.6% to 38.6% according to our results and other studies. Furthermore, findings suggest that older diabetic patients exhibit a greater prevalence of anemia compared to the general elderly population^42,44^. Older diabetic patients with anemia have more comorbidities than older diabetic patients without anemia^44^. Additionally, older patients with diabetes and comorbid anemia exhibit a higher incidence of hospitalization, increased frequency of medical interventions, and elevated mortality risk^44^. Therefore, it is crucial to employ effective interventions aimed at reducing the likelihood of anemia in elderly diabetic patients, as this may potentially improve patient outcomes.

### Relationship between serum ferritin, anemia, and hematological parameters

In older adults, iron, folate, and/or vitamin B12 deficiencies are responsible for about one third of anemia cases^45^. Nearly half of all nutrient deficiency-related anemia cases are cause by iron deficiency. The serum ferritin levels correlate closely with body iron stores, and its levels may serve as an indirect indicator of the overall iron reserves in the human body^9^. Several reports indicated that a low level of ferritin may indicate iron deficiency anemia^28–30^. The WHO defines low ferritin as levels < 15 μg/L for older persons (60 + years)^9^. In clinical settings, serum ferritin levels (with a cut-off of 30 g/L) are most useful in identifying iron deficiency, which exhibit superior sensitivity and specificity^46–48^. A serum ferritin level ranging from 20 to 50 μg/L is regarded as a zone of uncertainty. According to several reports suggested that a low serum ferritin level is less than 40 μg/L^28–30^. The present study revealed a noteworthy increase in the percentage of anemia among elderly patients with diabetes who exhibit a low level of serum ferritin (< 40 μg/L) when compared to other subgroups. The percentage of anemia in the low, lower side within the reference range, higher side within the reference range and high serum ferritin levels were 50.0, 27.7, 20.5 and 24.2% (*p* = 0.025), respectively.

Numerous studies have demonstrated that low levels of ferritin may be linked to hematologic alterations, in addition to serving as an indicator of iron deficiency anemia^28–30,49^. However, reports are scarce on association between low ferritin levels and hematological parameters in older patients with diabetes. A community-based cohort study found that RDW negatively associated with ferritin levels and Hb among older adults^49^. A study showed that inverse correlations between platelet counts with serum ferritin and Hb in women with iron deficiency anemia^50^. Another study indicated that patients with more severe and hypochromic anemia had higher platelet counts among female with iron deficiency anemia and thrombocytosis^34^. In our study, Hb and serum ferritin levels were positively correlated, while RDW and platelet count were negatively correlated with serum ferritin levels. Additionally, individuals with low serum ferritin levels were found to have a higher risk of anemia and elevated RDW.

A possible explanation for this finding is that RDW are elevated in iron deficiency^33,51^, resulting in an effect of deregulation of red blood cells homeostasis due to both impaired erythropoiesis and RBC degradation^33^. Additionally, iron deficiency might stimulate megakaryopoiesis, which excite the formation and differentiation of megakaryocytes that produce large numbers of platelets, thereby promoting platelet production^50,52^. On the contrary, iron overload may have an inhibitor effect on platelet counts^50^. Indeed, the present study showed high serum ferritin (> 200) μg/L had lower platelet counts.

It has been identified as a significant public health issue that anemia is prevalent in the elderly population with type 2 diabetes^38–41^. The data at our disposal, along with a number of prior investigations, indicate that low ferritin levels may be linked to anemia, elevated RDW risk, and increased platelet count. Additionally, our data indicated that 64.3% had iron intake which was less than the DRIs (< 10 mg/day). Iron deficiency with anemia contributed to the higher RDW and platelet count can accelerate the development of cardiovascular diseases and increased mortality risk in non-diabetic and diabetic patients^12,53,54^. Therefore, serum ferritin had a predictive value for outcome of hematological variations in iron deficiency anemia. It is advisable to consider regular evaluation of serum ferritin levels for the management of diabetes in older patients*.* Additionally, it is imperative to closely monitor dietary habits to mitigate the risk of iron deficiency anemia.

### Relationship between serum ferritin and liver functional parameters

In accordance with the WHO, individuals who are 60 years of age or older are susceptible to iron overload when their serum ferritin levels exceed 150 μg/L for females and 200 μg/L for males^9^. Several reports suggested that high serum ferritin level was > 200 ng/mL^28–30^. Elevated levels of ferritin indicate an augmented accumulation of iron in the body, which may be linked to a heightened susceptibility to liver dysfunction and inflammation^14,15,20,55^. The findings of our study indicate a statistically significant positive correlation between serum ferritin levels and GOP and GPT levels. High serum ferritin levels (> 200 ng/mL) were found to be significantly associated with an increased risk of elevated GPT. A cross-sectional study has demonstrated a significant association between GPT and serum ferritin levels, indicating a potential correlation between elevated serum ferritin and liver dysfunction^56^. Furthermore, previous cross-sectional research has provided evidence of a positive relationship between elevated serum ferritin and advanced hepatic fibrosis^57^. A raised serum ferritin was found to be positively correlated with increased iron accumulation in the body, as well as heightened hepatic iron deposition in both the reticuloendothelial system and hepatocytes. The patients with an elevated serum ferritin also had higher GOT and GPT, and a lower platelet count^57^. This means increased total body iron storage may be association with liver dysfunction and inflammation.

This study and several previous studies infer that the elevated serum ferritin was association with liver dysfunction. A possible explanation for this finding is that an elevated ferritin may meant that increased body iron storage, which may play a role in oxidation stress, inflammation, and liver damage. Injured hepatocytes augmented release ferritin into the serum. Ferritin also is an acute phase reactant, which can be produced by proinflammatory cytokines^14^. Furthermore, even a little accumulation of iron in liver combined with other cofactors can elevate oxidative stress, as a result, necrosis of liver cells and activation of hepatic stellate cells as well as hepatic fibrosis^58^.

This study suggested that serum ferritin may be considered as another parameter of liver function assay, along with the GOT and GPT. Nevertheless, further studies should be essential to confirm the role of inappropriate iron storage and the meaning of elevated serum ferritin in elderly diabetic patients with liver dysfunction.

### Relationship between serum ferritin and metabolic parameters

Increasing evidences have been revealed that body has too much iron storage not only raises risks for insulin resistance and diabetes but also induces cardiometabolic disorders in nondiabetic and diabetic persons^59^. A study found a strong positive correlation between serum ferritin and both BMI and CRP^60^. A recent study indicates the visceral fat mass and hsCRP were increased significantly with increased serum ferritin^61^. Specifically, a controlled, cross-sectional, single-center study demonstrated a significant elevation in serum ferritin levels with increasing HbA1c levels in individuals diagnosed with type 2 diabetes^62^. Furthermore, the study found a strong positive correlation between serum ferritin levels and both HbA1c and FBG levels^62^. Moreover, a retrospective cross-sectional study indicated a positive association between hemoglobin and ferritin levels with both blood pressure and hypertension risk among older adults^63^. In this study, high serum ferritin levels were associated with obesity, high FBG, and high PBG. The higher side within the reference range of serum ferritin also showed a significantly higher risk of high FBG and high HbA1c. Nevertheless, our data found that no significantly between associated serum ferritin with hsCRP, triglycerides, HDL-cholesterol and BP.

Type 2 diabetes is regarded as an inflammatory condition^21^. Possible explanations for the association between iron and glucose metabolism include the physiological action of insulin, which results in increased uptake of various nutrients, including iron^8,14^. Factors such as weight gain, aging, and inflammatory conditions may contribute to hyperinsulinemia, which exacerbates this process and leads to increased iron deposition, ultimately worsening insulin resistance. The accumulation of iron may also affect insulin synthesis and secretion in the pancreas as well as interfere with the liver's insulin-extracting capacity, thereby affecting glucose metabolism^8,14^.

The findings of this study are in agreement with numerous prior studies, indicating a noteworthy correlation between serum ferritin levels and both obesity and glycaemia. Therefore, it is recommended to regular blood tests, maintain ideal body weight, avoid excessive alcohol and vitamin C, and appropriate dietary intake are essential for older diabetic patients, which may benefit glycemic control^36,64,65^. However, additional research is necessary to gain a more comprehensive understanding of the role of serum ferritin levels in the obesity and glycemic control in elderly individuals with diabetes.

### Exploring of optimal thresholds for serum ferritin levels in older patients with diabetes

To date, although low serum ferritin indicates iron deficiency and an increase in serum ferritin levels is associated with liver dysfunction, obesity and not good glycemic control, it is unclear what cut-off points of serum ferritin for iron deficiency and overload iron or low-degree inflammatory condition in older persons with diabetes. The WHO has established that low ferritin levels are defined as being less than 15 μg/L, while levels exceeding 150 μg/L for females and 200 μg/L for males in individuals aged 60 years or older are indicative of a risk of iron overload^9^. In clinical settings, the most effective means of detecting iron deficiency is through the use of serum ferritin levels, with a cut-off value of less than 30 μg/L providing optimal sensitivity and specificity^46–48^. A serum ferritin level ranging from 20 to 50 μg/L is considered to be within a gray area. Previous reports have suggested that low serum ferritin levels are less than 40 μg/L, while high serum ferritin levels are greater than 200 μg/L^28–30^.

A cross-sectional study has demonstrated a noteworthy positive correlation between insulin resistance and serum ferritin in individuals with type 2 diabetes. Additionally, even when serum iron levels are within the normal range, they are still associated with insulin resistance^66^. Our findings suggest that individuals with low serum ferritin levels (< 40 μg/L) are at a higher risk of anemia and high RDW. Higher side within the reference range and high of serum ferritin levels, that is to say serum ferritin level was > 120 μg/L may be associated with liver dysfunction, obesity and not good glycemic control. Furthermore, our data revealed mean serum ferritin is approximately between 126.8 μg/L and 135.2 μg/L among patients with liver dysfunction, obesity or not good glycemic control. This implied the determination of normal ranges for serum ferritin may be too broad and the standard for iron overload may be too high. Serum ferritin is like a double-edged sword, which reveal that low or high levels is unfavorable to diabetic management in older adults. Further investigation is necessary to ascertain the specific serum ferritin thresholds indicative of iron deficiency, iron overload, or inflammatory conditions in elderly patients diagnosed with diabetes.

### Achievements and implications

The findings of our study have significant policy implications regarding the potential use of serum ferritin as a biomarker for hematologic alterations, liver function, obesity, and glycemic control, particularly among older adults with diabetes. Studies have shown that low serum ferritin levels can indicate iron deficiency anemia^28–30,49^. Elevated serum ferritin could suggest iron overload which increase the risks of liver dysfunction, obesity, and not good glycemic control has also been documented^60–62^. The cut-off points of serum ferritin for iron deficiency and iron overload in older diabetic patients are not well understood. This study has identified that the current determination of the normal range of serum ferritin may exhibit excessive breadth, while the established standard for iron overload may be excessively elevated. Consequently, additional investigations are imperative to ascertain the precise reference range for older patients with diabetes.

### Limitations and prospective

Our study has several limitations*.* Firstly, the cross-sectional study design made it impossible to establish an exact causal link between serum ferritin and anemia, liver function, and metabolic factors. Secondly, we employed the serum ferritin threshold established in prior research as a determinant of low (< 40 μg/L) and high (> 200 μg/L) serum ferritin levels. Nevertheless, the recommended cut-off values for serum ferritin remain a topic of ongoing debate, and it is believed that variations may not be accurately reflected in older adults with inflammatory conditions such as obesity and diabetes. The results of our study indicate that elderly diabetic patients with a serum ferritin level exceeding 120 μg/L exhibit a correlation with liver dysfunction, obesity, and suboptimal glycemic control. Therefore, future research is warranted to determine the precise cutoff points of serum ferritin for iron deficiency and iron overload or inflammatory condition in older patients with diabetes.

## Conclusions

Serum ferritin exhibits a dual nature, with levels below 40 μg/L indicating iron deficiency anemia and levels exceeding 120 μg/L indicating liver dysfunction, obesity, and suboptimal glycemic control. As such, serum ferritin may serve as a potential biomarker for assessing iron stores, liver function, and glycemic management in elderly patients with diabetes. Further investigation is necessary to determine the extent to which iron overload contributes to poor glycemic control and to establish an optimal threshold value for serum ferritin levels.

## Data Availability

The data are not publicly available due to privacy and ethical restrictions.
